# Colon-Specific Gut Microbiome Remodelling Is Associated with Host Metabolism in Paracetamol-Induced Liver Injury in Rats

**DOI:** 10.3390/metabo16100698

**Published:** 2026-09-22

**Authors:** Zhanagul Khassenbekova, Saniya Kozhakhmetova, Dinar Tarzhanova, Akmaral Zhantureyeva, Indira Uyzbayeva, Aruzhan Shoman, Tamara Tultabayeva, Damilya Konysbayeva, Kadyrzhan Makangali, Assem Sagandyk

**Affiliations:** 1 Innovative Center ArtScience, Astana 010000, Kazakhstan; saniya.s.kozhakhmetova@gmail.com; 2 Department of General Pharmacology, Non-Profit Joint-Stock Company “Astana Medical University”, Astana 010000, Kazakhstan; dinar.tsh@gmail.com (D.T.); zhantureeva.a@amu.kz (A.Z.); indira-2008@inbox.ru (I.U.); 3 The AgroTech Research and Innovation Center, Astana IT University, Astana 010000, Kazakhstan; a.shoman@astanait.edu.kz; 4 Institute of Engineering and Food Technology, Saken Seifullin Kazakh Agrotechnical Research University, Astana 010000, Kazakhstan; ttultabayeva@ucdavis.edu; 5 Department of Food Science and Technology, University of California, Davis, CA 95616, USA; 6 Institute of Agriculture and Forestry, Saken Seifullin Kazakh Agrotechnical Research University, Astana 010000, Kazakhstan; damilya_konysbaeva@mail.ru (D.K.); a.sagandyk@kazatu.edu.kz (A.S.); 7 Engineering Center for Organic Agricultural Technology, Saken Seifullin Kazakh Agrotechnical Research University, Astana 010000, Kazakhstan; k.makangali@kazatu.edu.kz

**Keywords:** gut–liver axis, microgreens, *Beta vulgaris*, *Artemisia dracunculus*, paracetamol, acetaminophen hepatotoxicity, gut microbiota, short-chain fatty acids, 16S rRNA, serum metabolomics

## Abstract

***Background/Objectives***: The gut and liver are functionally coupled along the gut–liver axis, yet most studies treat the intestine as a single compartment and sample feces alone. We tested whether a plant-derived beetroot–tarragon microgreen beverage engages this axis in a segment-selective, colon-anchored manner in a rat model of paracetamol-induced liver injury with the intestinal dysbiosis characteristic of this model. ***Methods***: In a rat model of paracetamol-induced liver injury with the intestinal dysbiosis characteristic of this model, three paracetamol-exposed groups—a paracetamol-only model (APAP) and two treatment arms receiving either the beetroot–tarragon microgreen beverage (APAP+MG) or a licorice-root decoction (APAP+LIC)—were compared against an intact control serving as a reference. The gut–liver axis was interrogated end-to-end by combining liver histology, serum ^1^H-NMR metabolomics and vitamin profiling, and 16S rRNA gene sequencing across five intestinal sites (duodenum, jejunum, ileum, colon, and faeces). Group differences were resolved with consensus differential-abundance testing, and colonic microbiota were linked to circulating metabolites through paired microbiome–metabolome integration. ***Results***: Paracetamol produced a reproducible sub-massive injury. Microgreens were associated with less histological injury, less weight loss, and a serum metabolome closest to the intact state among the treated arms, although no individual metabolite contrast survived FDR correction. Community separation localised to the colon as a genuine centroid shift (Aitchison PERMANOVA R^2^ = 0.22; PERMDISP non-significant), whereas small-intestinal separation partly reflected dispersion. The microgreen arm showed higher abundance of SCFA-associated Lachnospiraceae and lower *Escherichia–Shigella*, with a broader colonic response than licorice (27 vs. 4 differentially abundant genera). The microbiome–metabolome association was directional but exploratory; no genus–metabolite pair survived FDR correction. ***Conclusions***: The beverage was associated with attenuation of paracetamol-induced hepatic injury (assessed histologically only; serum ALT/AST were not measured) through a colon-localised, SCFA-oriented remodelling with a directional but non-significant shift of the serum metabolome toward the intact profile, extending the microbiome-modulating benefits of microgreens to an acute drug-injury setting and supporting their further development as functional beverages, subject to a dedicated safety evaluation. As a pilot study with modest sample sizes and non-time-matched group endpoints, these findings require confirmation in adequately powered, time-matched cohorts.

## 1. Introduction

The liver and gut microbiota are functionally coupled along the gut–liver axis, with microbial metabolism shaping hepatic homeostasis through immune-, metabolic-, and barrier-related pathways [1,2,3]. Depletion-and-restoration paradigms have shown that the trajectory of drug-induced liver injury depends on the resident community, establishing that the microbiota acts as a determinant rather than a correlate of hepatic outcome [4]. On this basis, microbiota-targeted interventions have been pursued as a strategy to attenuate liver injury, and several molecular routes have been described: resident *Lactobacillus* engage hepatic Nrf2 signalling to protect against oxidative damage [5]; microbial glycosidases liberate bioactive aglycones from dietary precursors with direct hepatoprotective activity [6]; *Akkermansia muciniphila* and other colonic anaerobes rescue community structure and short-chain fatty acid (SCFA) output following injury [7]; and polyphenol-rich phytochemical matrices remodel the microbiota and recalibrate host metabolism [8]. These findings identify the gut microbiota as a modifiable determinant of hepatic physiology.

However, several limitations remain. The intestine is often treated as a single compartment, and fecal sampling reduces a spatially structured ecosystem to a single readout. Yet pH, nutrient availability, bile acids, oxygen, and transit time all change along the gut and shape distinct microbial communities at different segments: the small intestine is dominated by facultative-anaerobic, proteolytic taxa, and the colon by obligate-anaerobic, saccharolytic ones [9]. Because colonic fermentation is the principal microbial source of metabolites reaching the systemic circulation [10], the anatomical site at which an intervention engages the microbiota directly determines whether, and how, it can influence the host. Group-level compositional differences are rarely separated from increases in between-animal variability, even though injury itself destabilises microbial communities and inflates apparent group effects. Paired microbiome–metabolome designs also remain uncommon and, when undertaken, typically involve sample sizes too small to resolve individual taxon–metabolite associations after correction for multiple testing. As a result, the gut–liver axis remains incompletely reconstructed, and the strength and directionality of its couplings are poorly quantified.

The present study employs a design that is simultaneously segment-resolved, multi-omic, and explicitly intended to discriminate directional community shifts from increases in between-animal variability, applied here to test a biogeographically grounded prediction about how a defined plant-derived nutraceutical engages the gut–liver axis. The intervention is a microgreen preparation from red beet (*Beta vulgaris* L.) and tarragon (*Artemisia dracunculus* L.), enriched in betalains, estragole, phenolic compounds, and flavonoid glycosides [11,12]. It combines fermentable substrates and conjugated polyphenols that largely escape proximal absorption and accumulate in the colon, where they are converted into short-chain fatty acids and bioactive aglycones. In the small intestine, by contrast, dense colonisation by rapidly dividing facultative anaerobes, predominantly *Helicobacter* and related *Campylobacterota* in rodents, together with rapid transit, narrows the ecological niche available for substrate-driven remodelling. These considerations predict that the action of the nutraceutical is not distributed uniformly along the gut but is anchored in the colon and propagated systemically through colonically derived metabolites.

We therefore test a single, pre-specified hypothesis: that the nutraceutical reshapes the gut microbiota in a segment-selective manner, with the dominant directional shift occurring in the colon and a more constrained response in the small intestine. This colonic remodelling should be coupled to a directed reconfiguration of the serum metabolome, including increased short-chain fatty acids and partial reversal of injury-associated amino-acid alterations, toward the uninjured state. Consequently, cross-omic structure across paired colon–serum samples should identify the colon as the principal anatomical interface through which the intervention engages the gut–liver axis.

Here, we examine how microbial communities are reshaped along the gut and how these changes relate to the serum metabolome in a rat model of drug-induced liver injury. We then test whether the action of the developed microgreen product is segment-selective and substrate-dependent, as predicted above. By combining five intestinal sites, the serum metabolome and vitamin status (the latter itself shaped by gut microbiome composition [13]) in a single design, our work offers a quantitative framework in which the gut–liver axis can be traced end-to-end and the contribution of a dietary intervention localised to a specific anatomical compartment.

## 2. Materials and Methods

### 2.1. Test Substance and Comparator Preparation

The plant material, microgreens of red beet (*Beta vulgaris* L.) and tarragon (*Artemisia dracunculus* L.), was supplied by the Astana IT University (Astana, Kazakhstan). It was grown under controlled vertical-farming conditions (22 ± 1 °C; 85% relative humidity; 16 h light/8 h dark; photosynthetic photon flux density 220 μmol m^−2^ s^−1^; sterilised peat-based substrate without nutrient supplementation) and harvested 12 days after germination, at full cotyledon expansion and emergence of the first true leaves. The botanical identity, growth protocol, proximate composition, full UHPLC-Q-ToF-MS phytochemical profile, total phenolic, flavonoid, betalain and chlorophyll content, and in vitro antioxidant activity (DPPH, ABTS, FRAP) of this material were characterised in detail by the same group and reported separately [12]. The principal bioactive markers are betalains, mainly betanin (82.45 ± 1.12 mg/100 g fresh weight), in beet microgreens, and estragole (34.18 ± 0.54 mg/100 g) with quercetin and apigenin glycosides in tarragon microgreens, plus hydroxycinnamic acid derivatives and flavonol glycosides in both species. Mineral and trace-element content of the same material was measured by ICP-OES (iCAP 6300 Duo, Thermo Fisher Scientific, Cambridge, UK); its vitamin content was profiled by ^1^H-NMR (JEOL JNM-ECA 500, 500 MHz JEOL Ltd., Tokyo, Japan), with the plant extract homogenised in D_2_O, clarified by centrifugation, adjusted to pH 6–7, referenced to sodium trimethylsilylpropionate (TSP; 0 ppm) and acquired with water suppression over 128 scans. Full mineral, trace-element, and vitamin values are reported in Supplementary Methods.

**Test substance and dosing suspension.** The microgreen substance administered in vivo was the pasteurised beetroot–tarragon microgreen beverage developed and characterised by the same group [14]. Briefly, freshly harvested microgreens of *B. vulgaris* and *A. dracunculus* were washed, ground to a paste, and extracted with 40% (*v*/*v*) ethanol at a plant-material-to-solvent ratio of 1:3 (*w*/*v*) under magnetic stirring for 1 h at room temperature followed by 24 h in the dark; the filtrates (Whatman No. 1) were concentrated on a rotary evaporator (Stegler; 30 °C) until complete removal of ethanol, giving beetroot and tarragon extracts of ≈7.6% and ≈7.0% total solids, respectively. The beverage was formulated (per 100 g of product) from 8 g of beetroot microgreen extract, 4 g of tarragon microgreen extract, 4 g of fructose, 4 g of citric acid, 0.2 g of ascorbic acid (vitamin C), and 79.8 g of water (≈10% total solids), homogenised, pasteurised at 65 ± 2 °C for 15–20 s, and cooled to 4 ± 2 °C. Each animal in the APAP+MG arm received 1 mL/day of this beverage by intragastric gavage over the 14-day intervention. One millilitre delivers ≈8.9 mg of microgreen-derived solids (≈0.61 mg from the beetroot and ≈0.28 mg from the tarragon extract), together with ≈40 mg fructose, ≈40 mg citric acid, and ≈2 mg ascorbic acid. Referred to the mean body weight of the group (≈0.25 kg), this corresponds to ≈35 mg of microgreen solids/kg/day, with ≈160 mg/kg/day of fructose, ≈160 mg/kg/day of citric acid, and ≈8 mg/kg/day of ascorbic acid co-administered.

**Licorice comparator.** Licorice (*Glycyrrhiza glabra*) root was chosen as a mechanistically informative contrast. Its principal constituent, glycyrrhizic acid, is a well-characterised hepatoprotectant in paracetamol injury, acting by suppressing TNFα-mediated hepatocyte apoptosis [15]. The two preparations are deliberately opposed: licorice acts through a single dominant principle, whereas the microgreen extract delivers a broad pool of fermentable substrates and phytochemicals. Licorice therefore serves as a narrow, single-principle reference and an established plant-based benchmark, against which the breadth and substrate-dependence of the microgreen response can be judged. The comparator given to the APAP+LIC arm was an aqueous decoction of pharmacopoeial-grade dried *G. glabra* root, standardised for glycyrrhizic acid per the State Pharmacopoeia of the Republic of Kazakhstan (monograph Glycyrrhizae radix). The root was decocted at 1:10 (*w*/*v*) in boiling water for 30 min, cooled to room temperature, and filtered. The clarified decoction was given by intragastric gavage at 1 mL/animal/day over the same 14-day window.

### 2.2. Animals, Hepatitis Model, and Interventions

The study used 38 outbred albino Wistar rats of both sexes, about 3 months old (≈12 weeks; initial body weight 180–360 g), housed under standard conditions (20 °C, 12 h light/12 h dark, food, and water ad libitum). All procedures were approved by the Innovation Center ArtScience Ethics Committee (protocol No. 6-2023, 30 May 2024) and followed Directive 2010/63/EU and the ARRIVE 2.0 guidelines [16].

In this experiment, we used a model of paracetamol-induced liver injury with concomitant intestinal dysbiosis. Drug-induced hepatitis with intestinal dysbiosis was induced with paracetamol, given intragastrically as a starch suspension at 1500 mg/kg in five doses on alternate days over 10 days; CON received the starch vehicle only. This dose, route, and schedule reproduce the sub-massive paracetamol hepatitis model of Pashko et al. [17], which reliably produces a mild-to-moderate hepatotoxic phenotype: elevated serum ALT (+96%), AST (+181%), γ-GT (+42%), and alkaline phosphatase (+67%), predominantly conjugated hyperbilirubinaemia, and inhibition of hepatocyte succinate and lactate dehydrogenases, against a background of focal vacuolar dystrophy, sinusoidal congestion and lympholeukocytic infiltration, with preserved lobular architecture. The model has also been characterised for its caecal microbial response under longer dosing [18]: acetaminophen markedly suppresses caecal lactobacilli and bifidobacteria with concomitant hepatomegaly and nephromegaly, providing direct precedent for the gut–microbial component examined here.

Paracetamol (acetaminophen) is among the most widely used analgesic–antipyretic agents and a first-line over-the-counter option for pain and fever, including in pregnant women and infants, with more than 50% of pregnant women worldwide reporting its use during gestation [19,20]. At therapeutic doses, it is largely safe: most is cleared by glucuronidation and sulfation, and only a small fraction is metabolised by cytochrome P450 (predominantly CYP2E1) to the reactive electrophile N-acetyl-p-benzoquinone imine (NAPQI), which is detoxified by conjugation with reduced glutathione. In overdose or with cumulative exposure, the conjugation pathways saturate, NAPQI accumulates, depletes mitochondrial glutathione, and, by covalent binding to mitochondrial proteins, drives oxidative stress, mitochondrial dysfunction, and centrilobular (zone 3) hepatocellular necrosis [21]. Accordingly, paracetamol remains the leading cause of acute drug-induced liver failure in the developed world [22]. This clinical relevance, reproducibility, and well-defined mechanism motivated the model. We used a high-dose regimen (1500 mg/kg) that reproduces a toxic, not a therapeutic, exposure.

Animals were allocated to four groups: intact control (CON; *n* = 7); paracetamol-induced hepatitis model (APAP; *n* = 10); and two treatment arms receiving paracetamol plus the microgreen product (APAP+MG; *n* = 11, of which two females died during terminal anaesthesia at the endpoint, from a presumed anaesthesia-related complication; no postmortem examination was possible, so a contribution of the intervention cannot be formally excluded, and both were excluded because a sampling complication then prevented adequate tissue and blood collection, leaving *n* = 9), or paracetamol plus the licorice comparator (APAP+LIC; *n* = 10).

From the start of the intervention, the APAP+MG and APAP+LIC groups received 1 mL/day intragastrically for 14 days. APAP+MG is the beetroot–tarragon microgreen beverage (*Beta vulgaris* L. + *Artemisia dracunculus* L.; see Section 2.1), and APAP+LIC is a decoction of licorice (*Glycyrrhiza glabra*) root. Endpoints were staggered: CON and APAP were terminated after induction, the treatment arms after the intervention. Body weight was recorded at baseline and at endpoint.

### 2.3. Sample Collection

At endpoint, animals were euthanized, and the following were collected from each: whole blood for serum; intestinal contents/mucosa from the duodenum, jejunum, ileum, and colon, plus faeces, for microbiota profiling; and liver and intestine for histology. Samples were snap-frozen and stored at −80 °C.

### 2.4. Liver Histology

Liver samples were fixed in 10% neutral-buffered formalin for 24–48 h at room temperature, dehydrated through graded ethanol, cleared in xylene, and paraffin-embedded. Sections were cut at 4 µm and stained with haematoxylin and eosin. Slides were examined by light microscopy at ×100–×400 magnification by a consultant pathologist blind to group allocation. Injury was graded semi-quantitatively on five domains: hepatocellular dystrophy, coagulative/lytic necrosis, sinusoidal/central-vein congestion, haemorrhage, and portal/perivascular inflammation, each scored 0–3 for a composite of 0–15 (Table 1). Because the consultant pathologist provided a single consensus assessment per group rather than per-animal blinded scores, per-domain and composite scores are reported as group-level semi-quantitative values (one score per group) and interpreted descriptively; per-animal inferential tests (e.g., Kruskal–Wallis) were therefore not applicable.

### 2.5. Serum Metabolomics and Vitamins

Serum metabolites and vitamins were quantified from the same ^1^H-NMR spectra, recorded on a JEOL JNM-ECA 500 spectrometer JEOL Ltd., Tokyo, Japan)) operating at a ^1^H frequency of 500 MHz (11.75 T; self-shielded compensated-solenoid magnet). Spectra were acquired in D2O with TSP as the chemical-shift reference and quantification standard, a relaxation delay (d1) of 5 s, and 16 scans; re-acquisition with 128 scans gave essentially identical results (within 0.05%). Because the signal-to-noise ratio increases approximately with the square root of the number of scans, 16 scans are adequate for the major, relatively high-abundance serum metabolites but do not, by themselves, demonstrate sufficient sensitivity for the direct quantification of low-abundance vitamins. The protocol was developed primarily for serum metabolomic profiling and is not a validated assay for the trace vitamins A and B12. Accordingly, the serum-vitamin values reported as unvalidated, profiling-grade relative intensities rather than calibrated concentrations, and vitamin A is not presented as a primary finding. Serum proteins were first removed by methanol precipitation: serum was mixed with methanol at 1:2 (*v*/*v*), vortexed for 30 s, and incubated at −20 °C for 20 min, and precipitated protein was pelleted by centrifugation (~13,000× *g*, 30 min). The supernatant was transferred to a fresh vial, dried in a vacuum concentrator (~3 h), reconstituted in 600 µL of deuterium–oxide (D_2_O) buffer containing 25 µmol of sodium trimethylsilylpropionate (TSP) as internal standard and chemical-shift reference (0 ppm), briefly re-centrifuged (~13,000× *g*, 5 min) and transferred to a 5 mm NMR tube. Spectra were acquired with a single-pulse ^1^H sequence (spectral centre 5.0 ppm; sweep width 9.384 kHz; 65,536 data points; digital resolution 0.143 Hz; 16 scans; relaxation delay 5 s; acquisition temperature 18.6 °C), then phased, baseline-corrected, and referenced to TSP. Characteristic signals for each analyte were integrated and converted to absolute concentrations against the TSP integral (internal-standard method), with a reproducibility of RSD ≤ 7% across quality-control replicates. Twenty-eight metabolites were quantified in this way. From the same spectra, six serum vitamins (A, C, D, B_1_, B_6_, and B_12_) were quantified as an independent analytical block; these serum vitamins are distinct from the vitamins profiled in the microgreen test substance (Section 2.1). Metabolite data were available for 29 of 38 animals (CON 7, APAP 5, APAP+MG 9, APAP+LIC 8); the missing samples reflect the limited blood/serum volume obtained at collection, which was insufficient for NMR in some animals, and the two missing APAP+MG samples correspond to the two females that died (Section 3.1).

### 2.6. 16S rRNA Gene Sequencing

Microbial DNA was extracted with the ZymoBIOMICS DNA Miniprep Kit (Zymo Research, D4300, Irvine, CA, USA). RNase-treated genomic DNA (≥30 ng per sample) was submitted to BGI Tech Solutions (Shenzhen, China), where the V3–V4 region of the 16S rRNA gene was amplified with 16S fusion primers from 30 ng of purified template DNA. The amplicons were purified with Agencourt AMPure XP magnetic beads (Beckman Coulter, Brea, CA, USA) and quantified on an Agilent 2100 Bioanalyzer (Agilent Technologies, Santa Clara, CA, USA). Qualified libraries were sequenced as 2 × 300 bp paired-end reads on the DNB platform (DNBSEQ-G400, MGI Tech. Shenzhen, China), with a nominal output of approximately 50,000 tags per sample delivered in FASTQ format. BGI performed sequencing and primary read filtering only; all taxonomic assignment and downstream analyses were carried out in-house. During primary filtering, raw reads were cleaned by removing primer and adapter contamination (cutadapt v2.6), low-quality sequences (Phred score < 20 over a 30-bp sliding window, reads retained only if ≥75% of their original length remained), reads containing ambiguous bases, and low-complexity reads (≥10 consecutive identical bases). Paired-end reads with sufficient overlap were then merged into consensus sequences with FLASH (minimum overlap 15 bp, mismatch ratio ≤ 0.1). The resulting clean reads were demultiplexed and processed with the sdm module of LotuS2 v2.23 [23], then denoised with the DADA2 algorithm and, after chimera removal, resolved into amplicon sequence variants (ASVs); no fixed-identity (e.g., 97%) OTU clustering was applied. Taxonomy was assigned by LAMBDA-based least-common-ancestor classification against the SILVA SSU database (v138.1), reaching ≈99% annotation at the phylum level and ≈80% at the genus level. Feature tables were built from phylum to genus and at the ASV level, and a maximum-likelihood phylogeny was reconstructed with FastTree2 from a MAFFT multiple-sequence alignment. Because the V3–V4 amplicon gives limited species-level resolution, cross-group analyses were restricted to the genus level, with species assignments reported descriptively only. This pipeline has previously been applied by our group to large-scale 16S surveys of the Kazakhstani population [24].

### 2.7. Bioinformatic and Statistical Analysis

All analyses were run in Python 3.12 and R 4.3, using established packages (phyloseq, vegan, decontam, ropls, mixOmics). Sex and intestinal site were carried as covariates throughout, with animal as the clustering unit for replicate and repeated measurements.

Of 109 demultiplexed mucosal-luminal samples, all that passed quality filtering were carried into analysis (Model 37, Microgreens 36, Licorice 36), including technical replicates of the same animal × site where present; no samples were merged. Technical replicates were not treated as independent biological observations: the animal was used as the clustering unit, PERMANOVA permutations were stratified by animal, and all inferential tests were performed within a single site (one biological sample per animal per site). Because cross-group comparisons require a site sampled in all arms and feces were not obtained in the APAP model, the primary analysis was restricted to the four mucosal-luminal sites (duodenum, jejunum, ileum, colon); the fecal layer was analysed separately among the treatment arms. As ASVs were inferred in independent per-group LotuS2 runs and are not comparable across groups, all cross-group analyses used genus-level tables (features present in ≥10% of samples within at least one site). Total sum-scaled relative abundances were used for compositional summaries, as well as centred log-ratio (CLR) values for ordination and parametric modelling.

Because negative controls and DNA-quantitation data were unavailable, decontam could not be applied [25]. Instead, putative contaminant, environmental, and plant-associated taxa were removed using a curated blocklist combining reagent/laboratory contaminants [26] and soil/rhizosphere lineages, cross-checked against LotuS2-flagged candidates, while *Helicobacter*/*Campylobacterota* were retained as bona fide rodent commensals. All downstream analyses were run in parallel on raw and filtered tables; main text numbers refer to the filtered table.

Alpha diversity (Shannon, observed richness) was compared across groups within each site by Kruskal–Wallis with Benjamini–Hochberg (BH) correction and pairwise Mann–Whitney U. Beta diversity used Aitchison and Bray–Curtis distances, with group separation tested by PERMANOVA (adonis2, 999 permutations; distance~group + sex, stratified by animal) and dispersion checked by PERMDISP. Samples from all groups were distributed across three concurrent sequencing runs on the same platform (DNBSEQ-G400) under identical library preparation and sequencing conditions and processed through a single pipeline. Sequencing run was therefore not confounded with group, and sequencing metrics were uniform across groups (Table S1). Differentially abundant genera in the colon were identified by a consensus of two complementary tests applied to three contrasts (APAP+MG vs. APAP, APAP+LIC vs. APAP, APAP+MG vs. APAP+LIC): a Mann–Whitney U test on log-relative abundances and a Welch *t*-test on CLR-transformed abundances. *p*-values from each test were Benjamini–Hochberg corrected within each contrast, and a genus was retained as differentially abundant only when it reached q < 0.10 in both tests (consensus intersection). Effect sizes are reported as log_2_ fold-change [27].

^1^H-NMR metabolite concentrations were log-transformed, autoscaled, and examined by PCA and OPLS-DA (ropls; 7-fold cross-validation, 999 permutations). Per-metabolite differences were tested by Kruskal–Wallis and, within four pre-specified contrasts, by Mann–Whitney U with BH correction; metabolic normalisation was defined both at the metabolite level (a shift opposite in sign to the injury vector, i.e., back toward the intact state) and at the animal level (Euclidean distance to the CON centroid). Vitamins were analysed as an independent block. In the 12 animals with paired colon-microbiome and metabolome data, integration used CLR-transformed colon profiles and autoscaled metabolites, with Spearman correlations (BH-FDR) and a permutation-tested PLS canonical axis; given the modest sample size, these analyses are reported as exploratory. Unless stated otherwise, significance was set at q < 0.05 after BH correction within each analysis family, all key analyses were repeated on the raw table as a sensitivity check, and the study follows the STORMS [28] and ARRIVE 2.0 guidelines.

## 3. Results

### 3.1. The Paracetamol Model Is Established: Microgreens Are Associated with Milder Somatic and Metabolic Manifestations of Liver Injury

Following the study design (Figure 1), paracetamol produced a reproducible hepatic injury: the model group developed the expected histological, somatic, and metabolic phenotype, clearly distinct from intact animals, confirming that the model was established. Both plant intervention arms, and the microgreen arm in particular, showed a milder phenotype. The microbial component of the model matches the precedent of Tropskaya et al. [18], who showed that 21-day intragastric acetaminophen in Wistar rats progressively depletes caecal lactobacilli and bifidobacteria while leaving the jejunal lumen essentially sterile, a spatial pattern that mirrors the colon-specific remodelling we describe below. Liver histology confirmed genuine injury, with visibly milder changes in the intervention arms; the spectrum of paracetamol-associated lesions is illustrated in Figure 2A–H, with composite and per-domain scores by group summarised in Table 1. Lobular architecture was preserved in all groups. Intact (CON) livers showed only diffuse hydropic and focally granular hepatocellular dystrophy, with intact nucleoli and no necrosis. The model showed the most pronounced changes: foci of focal coagulative necrosis, nuclear lysis and karyorrhexis, peri-portal haemorrhage, sinusoidal and central-vein congestion, perivascular lympholeukocytic infiltration, and moderate peri-central-vein fibrous proliferation. Both treatment arms were appreciably milder: the licorice group had fewer, smaller necrotic foci with regenerative features (binucleation, pseudoductules), and the microgreen group showed only rare scattered necroses, single portal lymphocytes, and a proportion of hepatocytes preserved, though a subset of surviving microgreen-treated females had marked central-vein congestion and sinusoidal oedema. Because the two deaths in this arm were presumed anaesthesia-related and occurred during the terminal procedure (Section 2.2), there is no direct evidence that these changes reflect intervention toxicity, although a treatment-related contribution cannot be formally excluded.

Condensing the consultant pathologist’s narrative into a five-domain rubric (necrosis, haemorrhage, congestion, inflammation, dystrophy; each 0–3) gave composite scores (0–15) of 4 (intact), 11 (model), 7 (licorice), and 6 (microgreens) (Table 1), a clear gradient of intact < microgreens ≈ licorice ≪ model. Because dystrophy was present in every group, including intact animals, the between-group separation was carried mainly by the necrosis, haemorrhage, congestion, and inflammation domains. The injury is thus best described as sub-massive. The pattern (preserved lobular architecture, focal coagulative necrosis, sinusoidal and central-vein congestion, perivascular infiltration, and vacuolar dystrophy) closely matches the sub-massive phenotype reported by Pashko et al. [17] and Radkovets et al. [29] at the identical dose, route, and schedule in Wistar rats, including the substantial inter-individual variability that motivated our semi-quantitative scoring. Serum ALT/AST and bilirubin were not measured in this cohort, so the biochemical magnitude is inferred indirectly from those reports, which document elevations in ALT (≈96%), AST (≈180%), γ-GT (≈42%), and alkaline phosphatase (≈67%) with predominantly conjugated hyperbilirubinaemia. The milder histology in both intervention arms, most clearly the microgreen arm, is therefore consistent with the lower serum-enzyme levels expected from these benchmarks; direct measurement of serum enzymes in a later cohort remains a priority.

At the organismal level, body-weight change differed across the arms with endpoint data (Kruskal–Wallis *p* = 0.027; Table 2): the model lost weight (median −26 g), microgreen-treated survivors essentially held their weight (+3.0 g; *n* = 9, after excluding two females that died), and the licorice group gained weight (+9.5 g). We did not measure fluid status or body composition; glycyrrhizin inhibits 11β-hydroxysteroid dehydrogenase type 2 and can cause pseudohyperaldosteronism with sodium and water retention [30,31,32], and weight gain on glycyrrhizin has previously been attributed to reversible fluid retention [33], so this interpretation is speculative rather than evidence of genuine recovery. The weight changes are, in any case, not directly comparable across groups because the follow-up intervals differ and the intact control has no endpoint weight. The model-versus-licorice contrast was significant (Dunn–BH q = 0.035); the model-versus-microgreens contrast stayed at trend level (q = 0.060).

The serum metabolome showed a consistent pattern (Figure 3). In the ordination, the model departed clearly from the intact state, while the microgreen metabolome lay closest to the intact centroid among the treated arms (median distance ≈6.6, versus ≈7.2 for the model and ≈7.8 for licorice; intact reference ≈4.5; Figure 3A). This reflected a robust injury signature in which glutamine, valine, leucine, tyrosine, glycine, histidine, glucose, creatinine, and acetate all differed from intact after correction, which microgreens shifted the profile in the normalising direction (19 of 28 metabolites; this directional count is not itself significant, binomial *p* ≈ 0.09; Figure 3B; Table S2). At the individual metabolite level, however, the microgreen-versus-model shifts were directional but did not survive correction: only acetate (a microbial SCFA; +1.16 log_2_ fold-change) and tyrosine (−1.09) reached trend level (q = 0.098), as expected given the limited power at *n* = 5 (model) versus 9 (microgreens).

Among the serum vitamins (Table 3, reported as unvalidated relative intensities rather than calibrated concentrations; see Section 2.5), vitamin A showed a directional pattern, lower with injury (model 6.8 versus intact 11.8) and higher with microgreens (9.5), but this is presented only as an exploratory observation and not as evidence of metabolic restoration. Vitamin D did not differ across groups (*p* = 0.469), and the rest moved in non-restorative directions: vitamin C was elevated by injury, and vitamin B_12_ was lower in the treatment arms, so the vitamin panel is reported in full rather than selectively.

### 3.2. The Gut Microbiome Shows a Pronounced Proximal-to-Distal Gradient with Structural Campylobacterota Dominance in the Small Intestine

We profiled the mucosal–luminal microbiota of the duodenum, jejunum, ileum, and colon across the three injury/intervention groups, with feces sampled in the treatment arms alone; the intact group served as a metabolome reference and was not sequenced. Of 109 demultiplexed mucosal–luminal samples (Model *n* = 37, Microgreens *n* = 36, Licorice *n* = 36), all 109 entered analysis (technical replicates retained but not treated as independent biological observations; see Section 2.7), alongside 23 fecal samples (Microgreens 8, Licorice 15). Sequencing depth was uniform across groups and sites: a median of 62,884 accepted reads per sample (IQR 62,294–63,786), with near-identical group medians (Model 62,878; Microgreens 62,858; Licorice 62,890), confirming comparable depth across arms (Table S1).

Libraries were sequenced to near-saturation: Good’s coverage reached ≈99.6–99.7% at every site in every group, so observed richness reflects biology rather than depth. A consistent proximal-to-distal richness gradient emerged across all three groups, at both ASV and genus levels. Small-intestinal samples (duodenum, jejunum, ileum) carried a median of ≈425–560 ASVs and ≈ 175–230 genus-level lineages per sample, colonic samples ≈975–1270 ASVs and ≈260–300 lineages, and faecal samples ≈1250–1270 ASVs and ≈290–300 lineages. In total, the dataset comprised 655 genus-level lineages (404 resolved genera) across the three groups.

This gradient coincided with a marked dominance: *Campylobacterota* (provisionally assigned to *Helicobacter*, species-level resolution still pending) made up ≈74–77% of reads in the duodenum, jejunum, and ileum, falling to ≈36% in the colon (Figure 4). This dominance is a structural property of the dataset, not a treatment effect. By compressing small-intestinal diversity, it motivated our use of CLR-transformed abundances, rather than raw proportions, for all compositional comparisons, with small-intestinal and colonic communities modelled jointly rather than pooled.

### 3.3. Community-Level Group Separation Localises to the Colon: A Clean Centroid Shift Versus a Dispersion-Mediated Signal in the Small Intestine

Alpha diversity (Shannon, genus level) followed the proximal-to-distal gradient (lowest in the small intestine, highest in the colon) yet did not differ between groups at any site (Kruskal–Wallis *p* = 0.56 in the duodenum, 0.09 in the jejunum at trend level, 0.20 in the ileum, 0.19 in the colon) (Figure S1) (Table 4). Colonic Shannon diversity was numerically far lower in the model (median ≈ 2.1) than in the treatment arms (≈3.7 in both; Figure S1). However, this came with considerable within-group dispersion (most in the Model and Licorice arms) and did not reach significance. We therefore report it as a non-significant trend, not a restoration of diversity.

In contrast, beta-diversity analysis showed significant group separation at every site (Aitchison PERMANOVA *R*^2^ = 0.15–0.22, *p* = 0.001–0.002), although it was strongest in the colon (*R*^2^ = 0.22) (Figure 5) (Table 4). With all sites pooled, the group factor explained 10% of the compositional variance (*R*^2^ = 0.10, *p* = 0.001). This PERMANOVA signal must be read alongside PERMDISP: dispersion was significantly heterogeneous in the ileum (*p* = 0.049) and borderline in the duodenum (*p* = 0.065), with the model arm more dispersed than the treated arms (Figure 5), so the small-intestinal separation partly reflects dispersion heterogeneity rather than a pure centroid shift. In the colon, by contrast, PERMDISP was clearly non-significant (*p* = 0.641); the colonic separation therefore represents a genuine community-state shift between the model and the interventions.

### 3.4. Differential Microbial Remodelling: A Broad Substrate-Driven Response to Microgreens Versus a Narrow Resident-Directed Response to Licorice

In the absence of negative controls, we applied a literature-informed taxonomic filter: 159 of 655 genus-level lineages were flagged as putatively environmental, reagent-associated, or plant-derived [26,34], together with chloroplast and mitochondrial 16S sequences and a curated set of environmental phyla unlikely to be true gut residents (Table S6). These flagged lineages were only 3.6% of all reads, the contaminant fraction being highest in the model group (3.84%) and slightly lower in the treatment arms (Microgreens 3.33%, Licorice 3.65%) (Table S3), consistent with the heavier handling burden of the model arm.

Consensus differential-abundance analysis on colon samples (requiring significance in both Mann–Whitney testing of log-relative abundances and Welch *t*-testing of CLR-transformed abundances, each BH-corrected and intersected at q < 0.10) identified 27 named genera separating APAP+MG from APAP after filtering (34 before; 7 contaminants removed), 4 separating APAP+LIC from APAP (4 before; the licorice signal was already gut-relevant and did not collapse on filtering), and 6 separating APAP+MG from APAP+LIC (8 before) (Figure 6 and Figure S2) (Table S4).

Microgreens enriched fibre-fermenting, SCFA-associated Lachnospiraceae taxa (Lachnospiraceae UCG-008, NK4A214 group, [Eubacterium] brachy group, [Ruminococcus] gauvreauii and torques groups, *Anaerostipes*, *Tyzzerella*, *Romboutsia*, *Flavonifractor*) together with *Prevotella* while depleting the pathobiont *Escherichia–Shigella*. The same signature, however, also included a fall in *Bifidobacterium* (a typically beneficial commensal) and reductions in *Subdoligranulum* and *Fusicatenibacter*. This is a mixed pattern we revisit in the Discussion, and one that argues against a uniformly beneficial remodelling. A further caveat is that several genera retained in the microgreens-versus-model set (*Kitasatospora*, *Methylocapsa*, *Dongia*, *Aurantimicrobium*) are environmental or plant-associated organisms. Because the intervention was administered as a plant extract, part of this signal may reflect the introduced plant matrix rather than genuine gut remodelling, so the number of confidently gut-resident differentially abundant genera is more conservative than the nominal 27. The licorice signal was narrower and gut-relevant by construction (the same Lachnospiraceae taxa elevated, no filtering-induced collapse), and the microgreens-versus-licorice differences were localised to a few taxa (most prominently *Kitasatospora*, *Acetitomaculum*, *Veillonella* and the [Ruminococcus] gauvreauii group, all higher in microgreens). Overall, after filtering, microgreens elicited a substantially broader gut-specific remodelling than licorice (27 versus 4 named differentially abundant genera in the colon) in keeping with the metabolome shift toward intact described in Section 3.1.

### 3.5. Microbiome–Metabolome Integration: Directional Support for a Gut–Liver Axis, Underpowered for Genus-Level Inference

Twelve animals provided paired colon mucosal–luminal microbiome (genus-level lineages) and complete serum ^1^H-NMR metabolome data: 5 Model, 4 Microgreens, 3 Licorice. Within this subset, FDR-controlled Spearman correlation tested 8960 genus–metabolite pairs (320 lineages × 28 metabolites, prevalence ≥ 30%); no pair survived the BH-FDR threshold (q < 0.10) before or after filtering (best q = 0.25 before, 0.29 after) (Table S5). A PLS shared-latent axis pointed to a high apparent cross-block correlation between microbiome and metabolome scores (r = 0.86), yet this fell short of significance against a permutation null (perm *p* = 0.13), and after filtering, both the correlation and its support weakened (r = 0.81, perm *p* = 0.37; Figure 7).

The directional consistency between the two data blocks therefore shows at the level of overall group separation: the model, microgreen, and licorice samples occupy distinct regions of the joint PLS plane (Figure 7). However, the small paired sample size precludes inference at the individual genus–metabolite level. Two of the highest-ranked, non-significant pairs (before and after filtering) are biologically plausible enough to flag as hypotheses for targeted validation: a strong positive *Escherichia–Shigella*–lysine correlation (rho = +0.90, *p* = 6 × 10^−5^, q = 0.25), consistent with the heightened *Escherichia–Shigella* signature of the Model arm and the elevated serum amino-acid pool of injury; and a negative Lachnospiraceae ND3007 group–alanine correlation (rho = −0.90, *p* = 8 × 10^−5^, q = 0.25), consistent with a fibre-fermenter/SCFA axis. The previously reported FDR-surviving *Curtobacterium* and *Delftia* signals did not survive the present recomputation (best q = 0.86 and 0.95); as both are environmental/reagent-associated and were flagged by the filter, this is reassuring. The integration analyses are therefore exploratory and underpowered at the current sample size, and the acetate–fibre-fermenter and *Escherichia–Shigella*–aromatic-amino-acid relationships are advanced as hypotheses for targeted validation rather than demonstrated links.

## 4. Discussion

In this paracetamol model of liver injury, the beetroot–tarragon microgreen beverage was associated with several concurrent differences: less histological damage, less weight loss, a serum metabolome closer to the intact state, and a gut community shifted in the colon toward short-chain fatty acid (SCFA) producers. Licorice acts through a single principal compound, whereas the microgreen produced a much broader colonic response, as expected from its many phytochemicals, which are part of what makes microgreens appealing as functional food ingredients.

The effect we see fits a growing body of microgreen research. In mice, red cabbage microgreens lower circulating LDL, hepatic cholesterol, and inflammatory cytokine expression, and they limit weight gain on a high-fat diet [35]. They also reshape the gut microbiota in ways tied to how the liver handles cholesterol, with one intake-responsive Clostridiales taxon tracking inversely with hepatic cholesterol ester [36]. Broccoli microgreen juice likewise improves insulin sensitivity, lowers the Firmicutes/Bacteroidetes ratio, raises SCFA-associated taxa, and reduces endotoxaemia in obese mice [37]. Earlier work focused on chronic metabolic or obesity settings. To our knowledge, ours is the first to show a microgreen benefit in acute drug-induced liver injury. This extends their reach beyond diet-related disease and echoes the protection that Brassica extracts provide against acute (lipopolysaccharide) liver injury through microbiota modulation [38].

The microgreen arm showed higher abundance of fibre-fermenting, SCFA-producing Lachnospiraceae together with Prevotella, as well as lower abundance of the pathobiont Escherichia–Shigella. This is the same functional signature (more SCFA producers) that underlies the Brassica-microgreen and broccoli-extract benefits above. Because we sampled five intestinal segments rather than feces alone, we could place the microgreen signal specifically in the colon, where it showed up as a genuine shift in community composition (a centroid shift). In the small intestine, by contrast, the apparent separation reflected mainly greater variability between animals (dispersion). Fecal-only studies cannot draw this distinction, and it points to the colon as the most likely site where the intervention engages the gut–liver axis.

The two ingredients act through complementary and converging mechanisms. Beetroot betalains and phenolics induce phase II detoxification and blunt the rise in transaminases and hepatic oxidative injury in rodents [39], while ethanolic tarragon (*Artemisia dracunculus*) extract, rich in caffeoylquinic acids and flavonoids, lowers malondialdehyde and other systemic markers of oxidative stress and suppresses NF-κB–p65 and NLRP3-inflammasome cytokines (IL-1β, IL-18) in rats [40]. Our results are consistent with this dual, antioxidant, Nrf2-oriented mode of action: the microgreen arm lay closest to the intact metabolome, with 19 of 28 metabolites moving toward the uninjured profile, although these shifts did not reach per-metabolite significance, and the serum-vitamin values are unvalidated. These betalain and phenolic antioxidants are delivered together in a single low-energy drink.

The link between microbiome and metabolome pointed in a consistent direction, even if it was underpowered: treated and model animals separated partly in the joint PLS space, and colonic taxa tracked amino-acid metabolites. This agrees with the microbiota-mediated hepatic effects reported for Brassica microgreens and extracts and is compatible with the hypothesis that SCFA and portal signalling link colonic remodelling to hepatoprotection, a hypothesis that remains to be tested in adequately powered studies.

Microgreens concentrate betalains and phenolics in a small serving, and the food-grade hydroalcoholic (“green”) extraction used here keeps this density of bioactives in a scalable, low-fat, low-energy beverage, a practical basis for developing it as a functional food.

**Limitations.** This was a pilot study: group sizes were modest, profiling was genus-level (V3–V4), and the paired integration cohort was small (*n* = 12). First, the intact control and the paracetamol model were sampled at the end of induction, whereas the treatment arms were sampled 14 days later. Because the study lacks a contemporaneous paracetamol-only endpoint and acute paracetamol injury resolves over time, spontaneous recovery cannot be separated from a treatment effect, and all treated-versus-model contrasts are therefore presented as associations. Second, serum ALT/AST and bilirubin were not measured and no serum remained for retrospective assay, so hepatoprotection was assessed histologically only; although the pathologist examined the slides blind to group allocation, scoring was reported at the group level (a single consensus assessment per group) rather than as per-animal scores, which precludes per-animal inferential statistics. Third, in the absence of negative controls, a literature-informed taxonomic filter was applied. Some biologically relevant taxa may have been removed, although the conclusions were stable before and after filtering, and the differential-abundance and integration results are reported as exploratory. Fourth, metabolite data were available for 29 of 38 animals, mostly missing from the APAP arm, which could bias the model metabolome, and the serum-vitamin values are unvalidated relative intensities. Because the beverage also delivered small amounts of ascorbic acid, fructose, and citric acid that were absent from the comparator arms, the serum vitamin C, glucose, and acetate signals in the microgreen arm may partly reflect these co-delivered constituents rather than microbiome-mediated effects. A matched-vehicle, dose-controlled study is the logical next step. Finally, the intact control group was not sequenced, so no healthy-baseline microbiota was available. The study therefore cannot establish paracetamol-associated dysbiosis or its correction, and all microbiome analyses are restricted to comparisons among the paracetamol-exposed arms.

## 5. Conclusions

A beetroot–tarragon microgreen beverage was associated with attenuation of paracetamol-induced hepatic injury (assessed histologically only; serum ALT/AST were not measured) and with a colon-localised, SCFA-oriented gut remodelling. The primary aim of the study was to characterise the intestinal microbiota under paracetamol exposure and to compare microbiota composition among the paracetamol-exposed arms. Because the intact control was not sequenced, the study cannot establish paracetamol-associated dysbiosis relative to a healthy baseline, nor its correction; the microbiome findings are limited to differences among the paracetamol-exposed arms. Given the pilot design, the non-time-matched group endpoints, and sex-specific histological changes in some surviving females, these findings require confirmation in adequately powered, time-matched cohorts and a dedicated safety evaluation before the beverage is developed as a functional food.

## Figures and Tables

**Figure 1:**
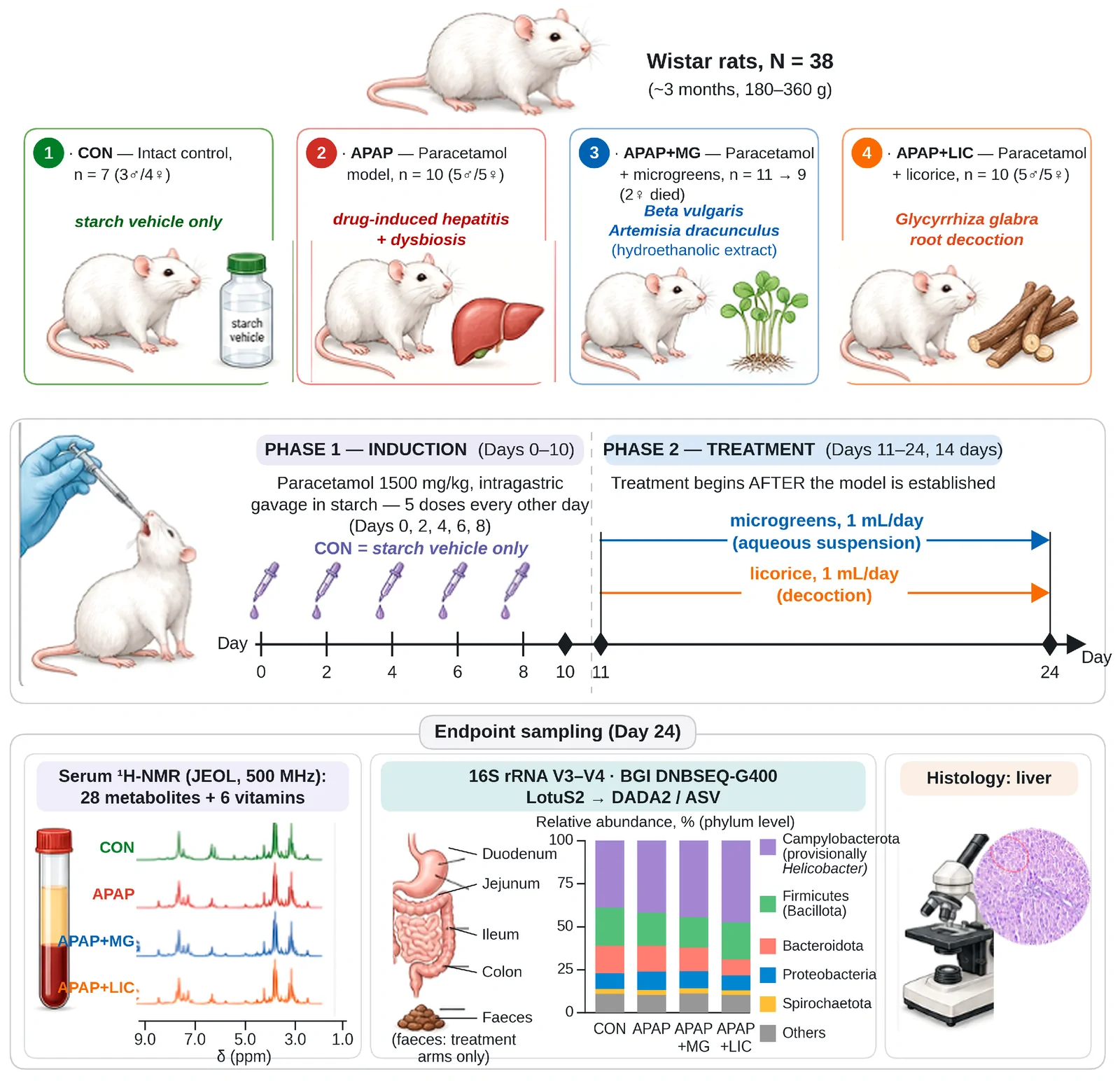
Study design. Thirty-eight Wistar rats were allocated to an intact control (CON), a paracetamol model (APAP) and two treatment arms receiving the beetroot–tarragon microgreen beverage (APAP+MG) or a licorice-root decoction (APAP+LIC). Paracetamol-induced liver injury with concomitant intestinal dysbiosis was established over 10 days and treatments were given for 14 days by intragastric gavage; liver, serum, and five intestinal sites were then sampled for histology, serum ^1^H-NMR metabolomics and vitamins, and 16S rRNA gene profiling.

**Figure 2:**
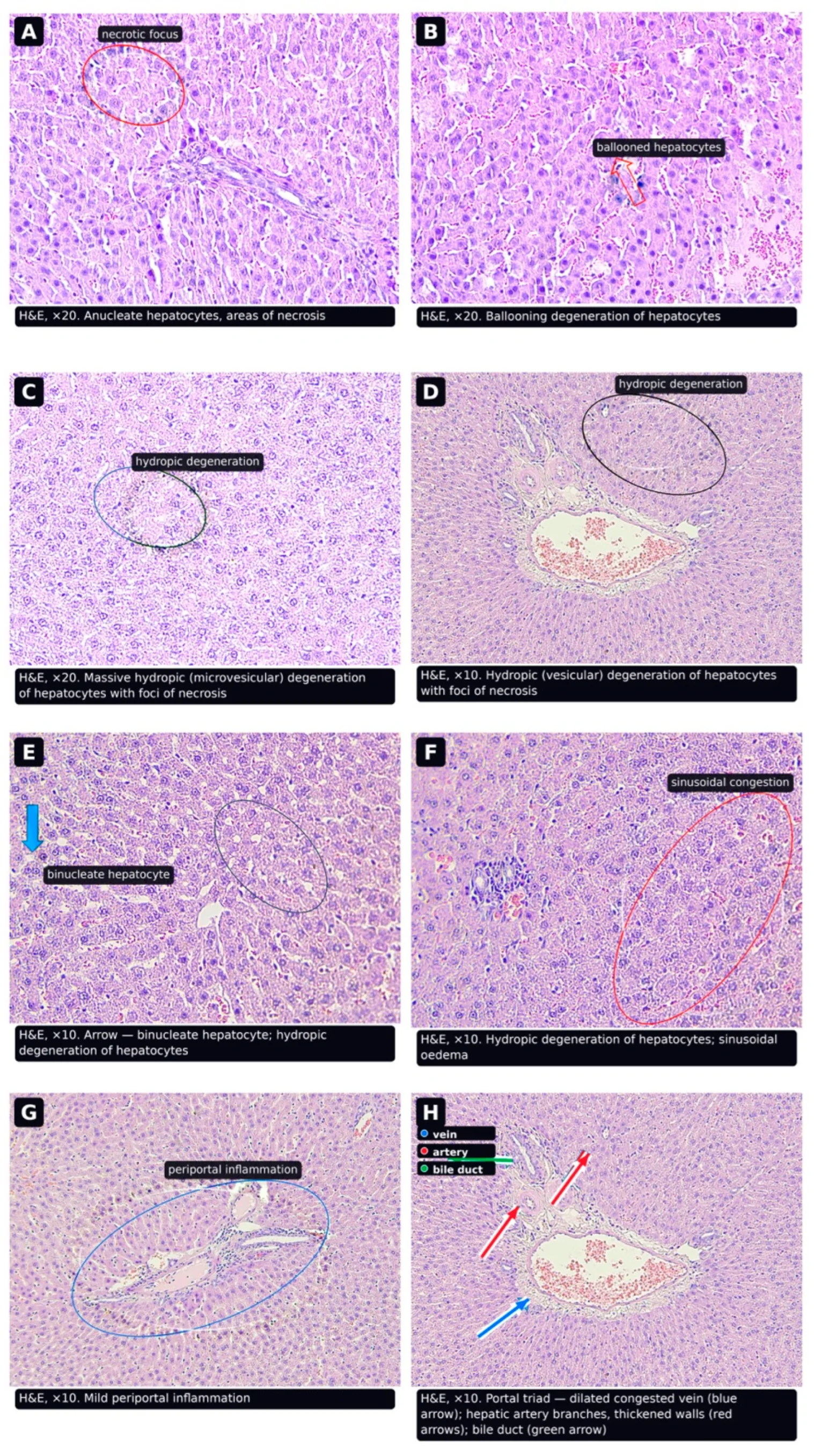
Representative liver histopathology in paracetamol-induced injury (haematoxylin–eosin), illustrating the spectrum of lesions scored in Table 1: (**A**) area of necrosis with anucleate hepatocytes (×20); (**B**) ballooning degeneration of hepatocytes (×20); (**C**) massive hydropic (microvesicular) degeneration with foci of necrosis (×20); (**D**) hydropic (vesicular) degeneration with foci of necrosis (×10); (**E**) hydropic degeneration with a binucleate (regenerative) hepatocyte (arrow) (×10); (**F**) hydropic degeneration with sinusoidal congestion and oedema (×10); (**G**) mild periportal inflammation (×10); (**H**) portal triad with a dilated congested vein (blue arrow), hepatic-artery branches with thickened walls (red arrows), and a bile duct (green arrow) (×10). Per-panel magnification and annotations are indicated on each micrograph; composite and per-domain scores by group are summarised in Table 1.

**Figure 3:**
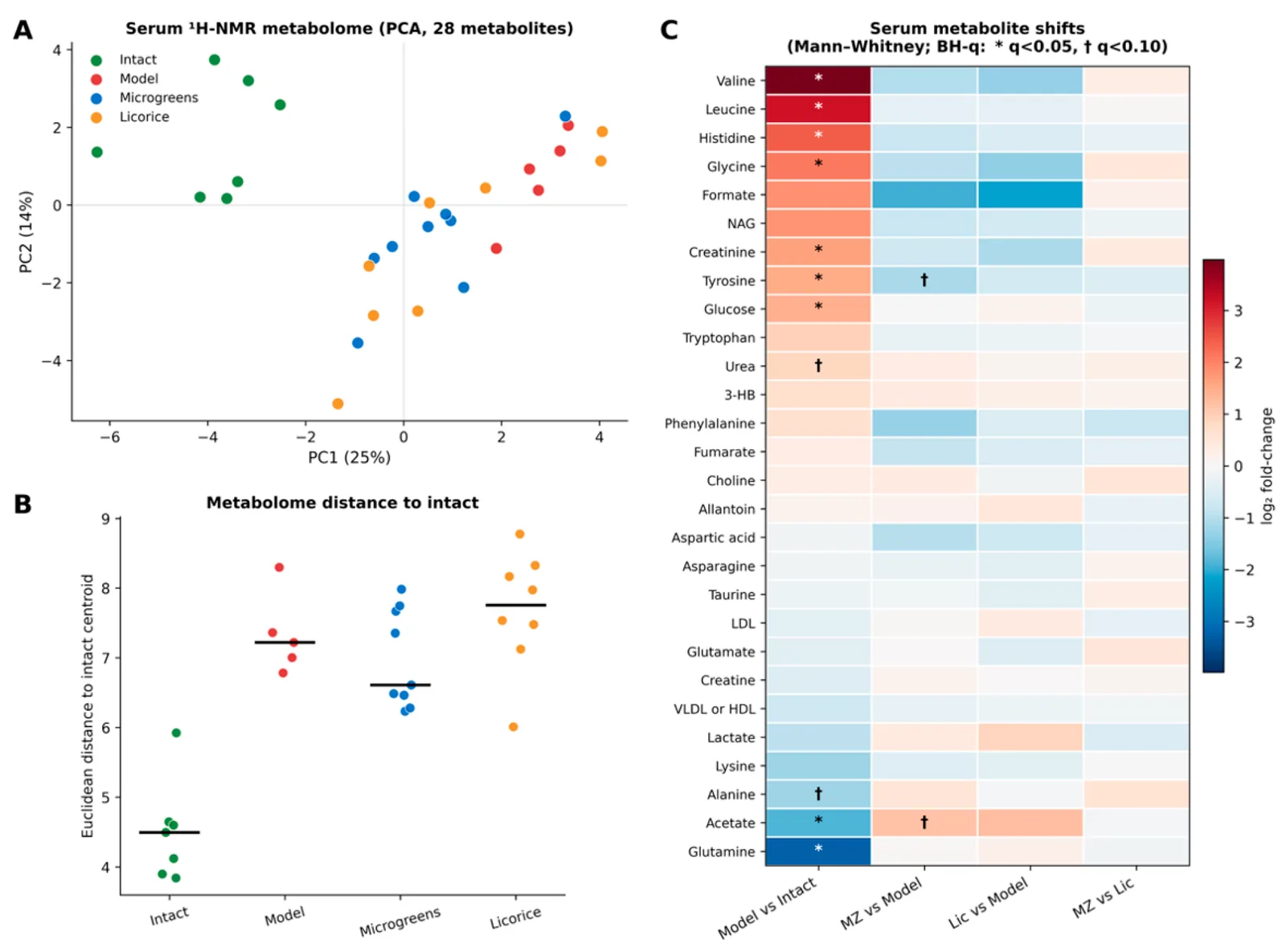
Serum ^1^H-NMR metabolome. (**A**) PCA of the serum metabolome (28 metabolites); each point is one animal, coloured by group (intact, CON; model, APAP; APAP+MG, microgreens; APAP+LIC, licorice). PC1 and PC2 explain 25% and 14% of the variance. (**B**) Euclidean distance of each animal to the intact (CON) centroid; bars are group medians. The distance was smallest for APAP+MG (≈6.6) among treated arms (model ≈ 7.2; licorice ≈ 7.8; intact ≈ 4.5), indicating a directional but non-significant shift toward the uninjured state. (**C**) Heatmap of metabolite shifts (log_2_ fold-change) for four contrasts (red, higher; blue, lower). Symbols denote Mann–Whitney significance after BH correction (* q < 0.05; † q < 0.10). Full values are given in Table S2.

**Figure 4:**
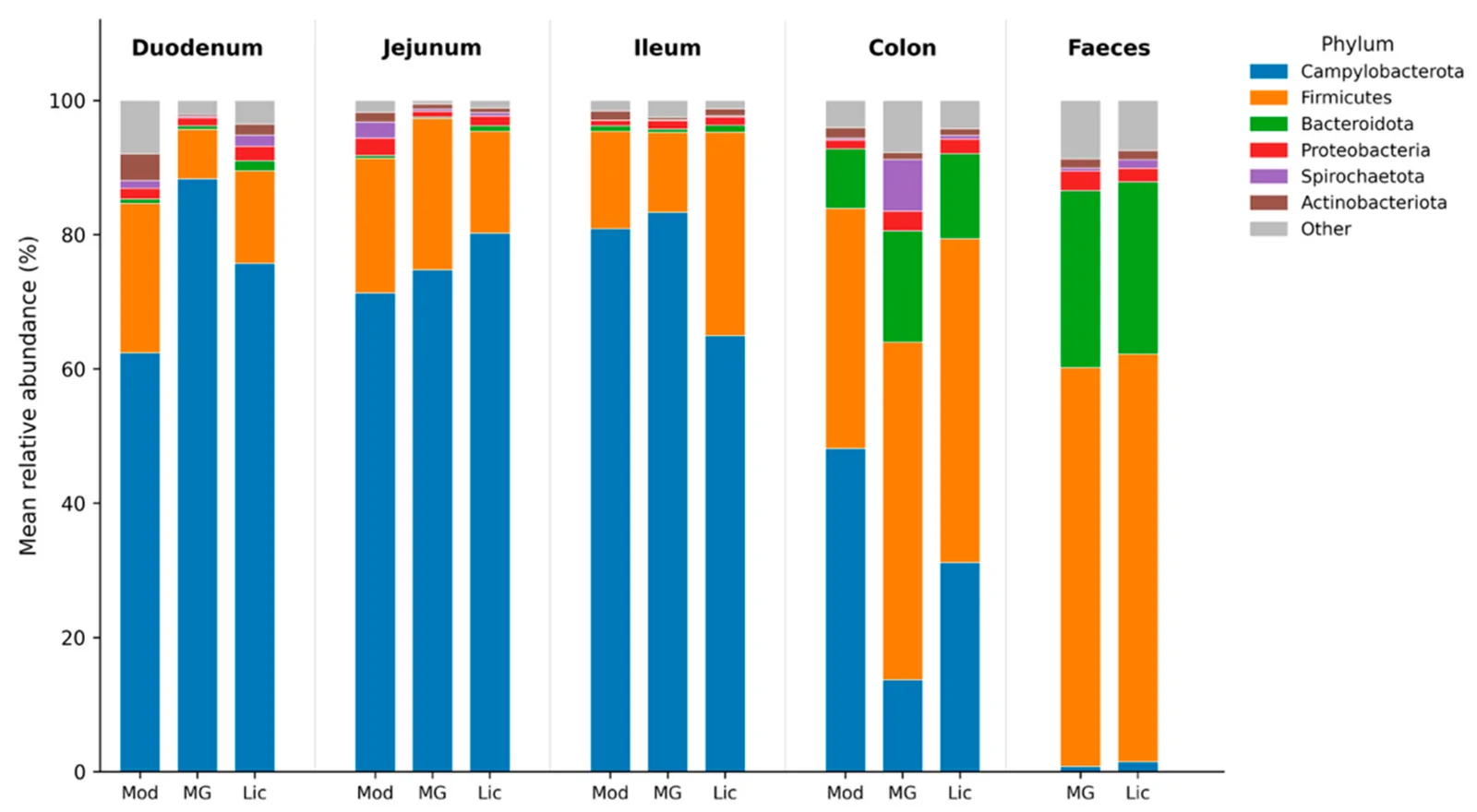
Phylum-level composition across the five intestinal sites, illustrating the proximal-to-distal gradient, with *Campylobacterota* comprising ≈74–77% of reads in the duodenum, jejunum, and ileum and falling to ≈36% in the colon.

**Figure 5:**
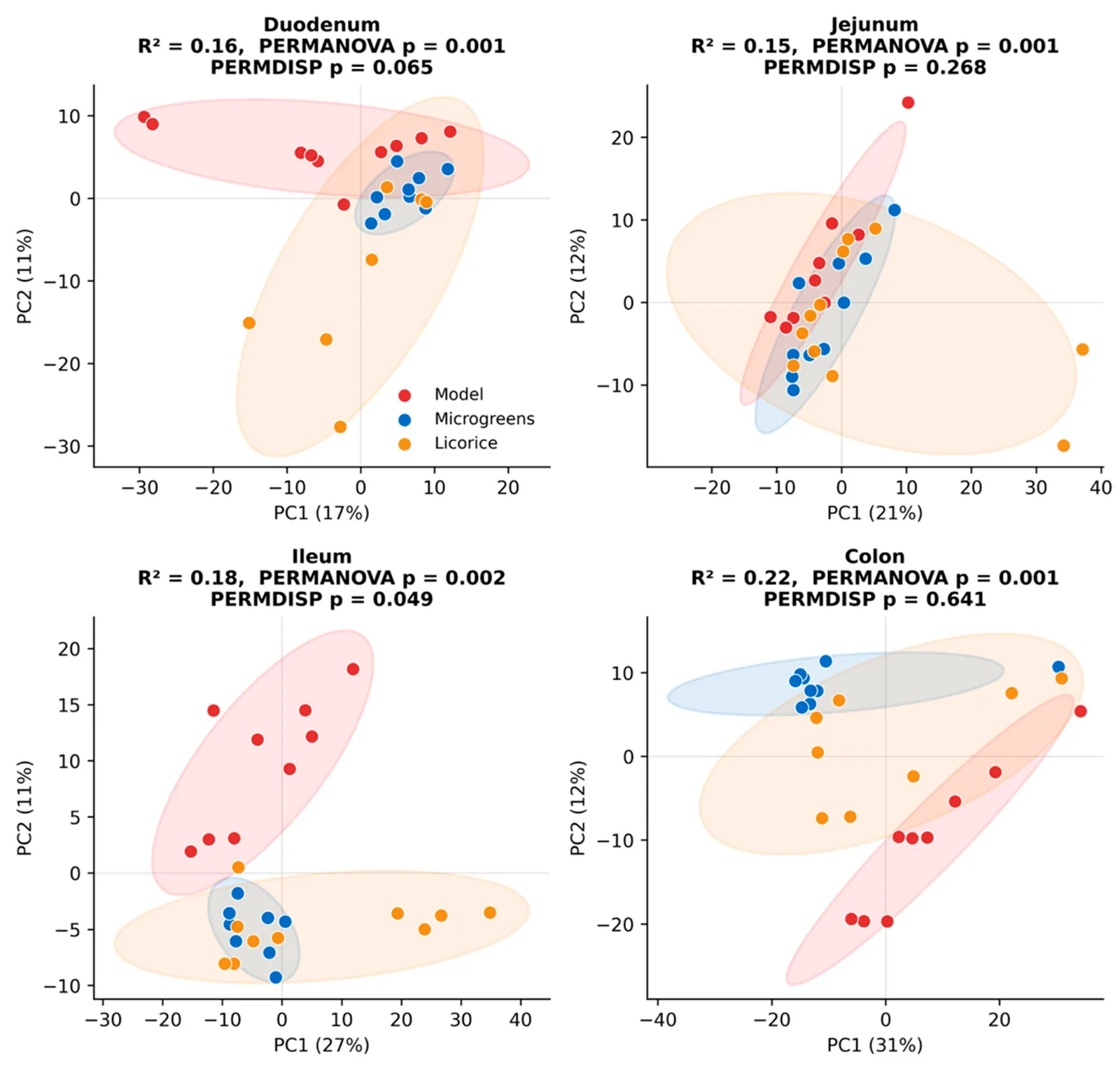
Beta-diversity ordination by intestinal site (Aitchison and Bray–Curtis distances) with PERMANOVA and PERMDISP results. Group separation was strongest in the colon (R^2^ = 0.22) and reflected a centroid shift rather than dispersion, whereas in the small intestine, it partly reflected greater dispersion of the model arm (Table 4).

**Figure 6:**
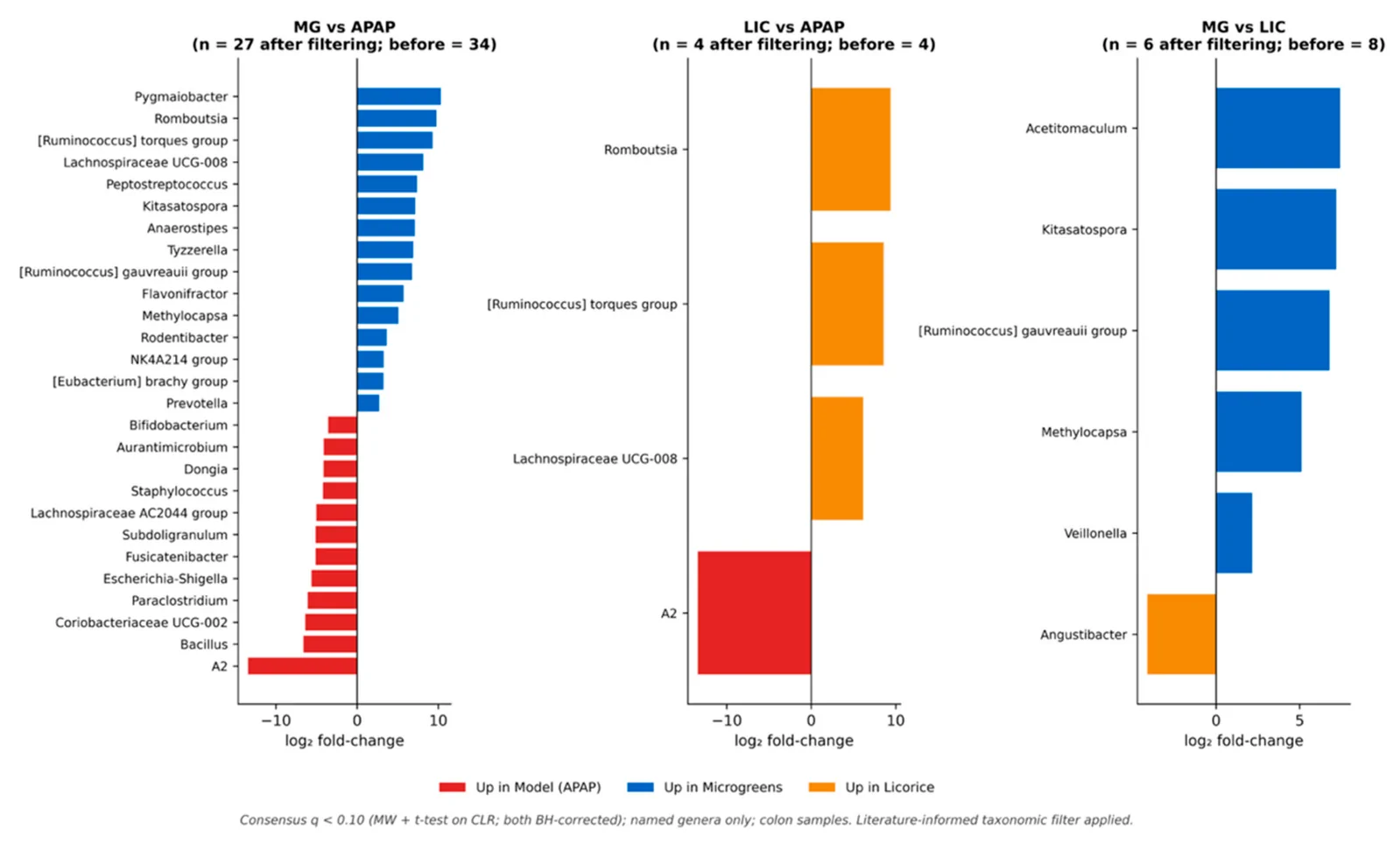
Consensus differentially abundant genera in the colon for the three contrasts (APAP+MG vs. APAP, APAP+LIC vs. APAP, APAP+MG vs. APAP+LIC) after literature-informed taxonomic filtering (consensus q < 0.10; bars show log_2_ fold-change). The effect of filtering on hit counts is shown in Figure S2 and the full list in Table S4.

**Figure 7:**
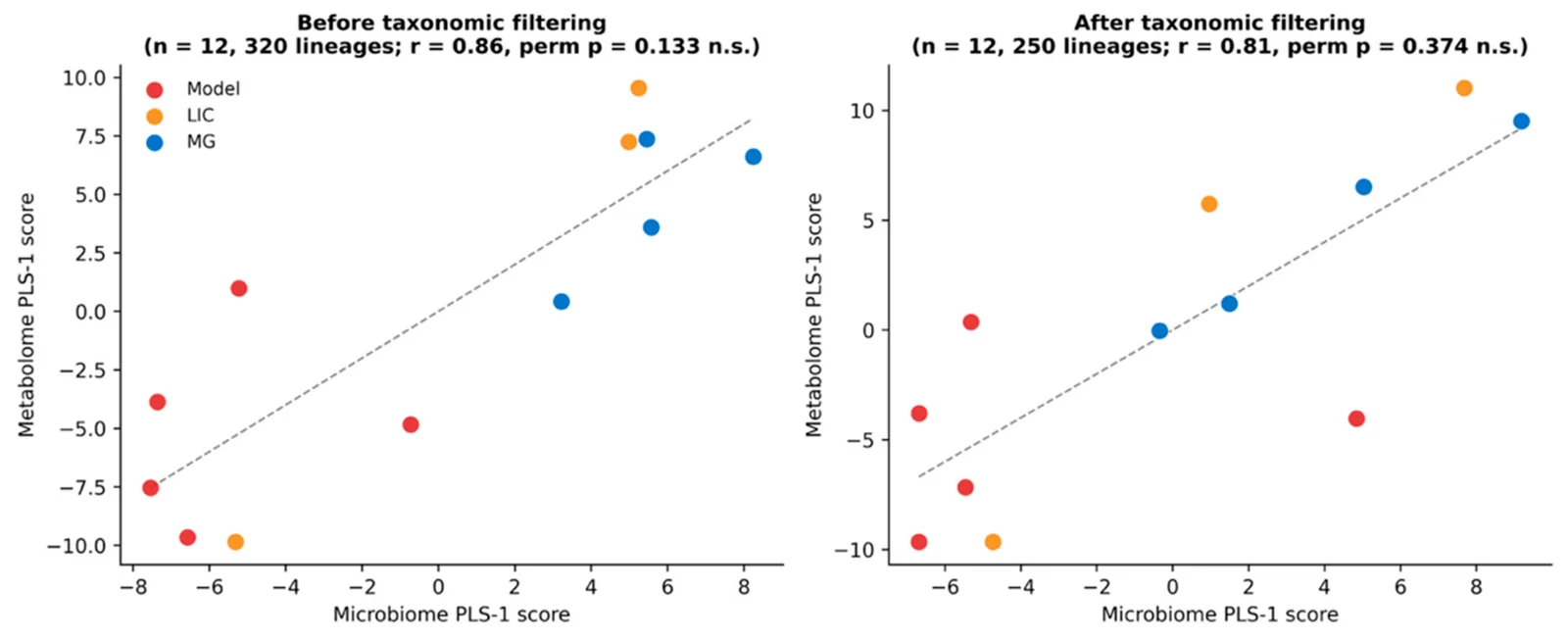
Microbiome–metabolome integration for paired colon samples (*n* = 12). Positions of individual animals in the joint PLS latent space; treated and model animals occupy partly distinct regions, but the small paired sample precludes firm inference, and these analyses are reported as exploratory (Table S5).

**Table 1:** Liver histology: semi-quantitative scoring and descriptive features.

Domain (0–3)	Intact (CON)	Model (APAP)	Licorice (APAP+LIC)	Microgreens (APAP+MG)	Pathologist’s Key Wording
Hepatocellular necrosis	0	2	1	1	Model: foci of focal coagulative necrosis. Licorice: single cells with nuclear lysis forming small necroses, also periportal. Microgreens: single small scattered necroses. Intact: none.
Haemorrhage	0	2	0	0	Model: small periportal haemorrhages. Licorice/MG/Intact: not described (only intra-sinusoidal erythrocytes).
Vascular congestion	1	2	2	2	Model/Licorice/MG: central veins moderately congested; sinusoids dilated with erythrocytes. Intact: central veins of normal diameter, mostly empty lumen.
Inflammation	1	2	2	1	Model: periportal + perivascular + perivenular lympholeukocytic infiltration, with peri-central-vein fibrous proliferation. Licorice: moderate periportal infiltration. MG: single periportal lymphocytes. Intact: single periportal lymphocytes (background).
Hepatocellular dystrophy	2	3	2	2	Model: hyaline-droplet dystrophy with blurred cell borders. Licorice: diffuse hydropic + focal hyaline-droplet (peri-central). MG: diffuse hyaline-droplet + single vacuolar; part of cells preserved. Intact: diffuse hydropic.
Composite score (0–15)	4	11	7	6	Gradient: Intact < Microgreens ≈ Licorice ≪ Model.

Scoring rubric: each domain 0–3 (0 = absent/normal; 1 = minimal or focal/single; 2 = moderate/multifocal; 3 = severe/diffuse/extensive). Composite = sum of the five domains (0–15). Derivation: these scores summarise the group-level qualitative description provided by the consultant pathologist, translated into a 5-domain semi-quantitative rubric. They are NOT per-animal blinded scores; per-animal inferential statistics cannot be reported on this basis. Note: hepatocellular dystrophy was present in every group, including intact; between-group separation is carried mainly by necrosis, haemorrhage, congestion, and inflammation. The injury is sub-massive (lobular architecture preserved throughout).

**Table 2:** Body weight change from baseline to endpoint, by group.

Group	*n*	Baseline, Median (g)	Endpoint, Median (g)	Change, Median (g)	Change IQR; Range (g)
Intact (CON)	7	293	—	n/a	terminated at induction (no endpoint)
Model (APAP)	10	279	239.2	−26	−51.8 to −17.0; −82.3 to +28.0
Licorice (APAP+LIC)	10	241	249.5	9.5	−6.5 to +19.5; −76.5 to +63.2
Microgreens (APAP+MG)	9	244.5	262.5	3	−7.5 to +15.0; −30.0 to +29.8

Omnibus: Kruskal–Wallis across the three arms with endpoint weights (Model, Licorice, Microgreens): H = 7.25, *p* = 0.027. Microgreens: two females died during the study (cohort 11 → 9 analysed) and were excluded from the weight-change analysis as deaths; the value among survivors is +3.0 g. Mortality (2/11) is reported separately. Caveat: body weight is a coarse severity index; the licorice gain (+9.5 g) plausibly reflects glycyrrhizin-associated fluid retention rather than recovery.

**Table 3:** Serum vitamins by group, median (IQR), unvalidated relative ^1^H-NMR intensities (arbitrary units); not calibrated concentrations (see Section 2.5).

Vitamin	Intact (CON)	Model (APAP)	Licorice (APAP+LIC)	Microgreens (APAP+MG)	KW *p*
Vit A	11.79 (9.62–18.66)	6.80 (6.56–8.19)	6.79 (6.38–8.72)	9.54 (7.68–10.47)	0.024 *
Vit C	5.84 (5.02–8.36)	20.60 (17.55–23.95)	11.42 (10.08–12.54)	15.47 (10.88–29.25)	0.013 *
Vit D	4.87 (3.27–7.40)	3.38 (3.24–5.68)	3.43 (2.81–4.23)	4.45 (3.24–4.76)	0.469
Vit B1	13.50 (7.17–20.39)	4.74 (4.50–11.22)	7.00 (4.50–12.22)	5.77 (2.39–8.86)	0.232
Vit B6	0.41 (0.26–0.83)	0.19 (0.18–12.47)	0.38 (0.22–2.32)	0.18 (0.13–0.56)	0.473
Vit B12	0.19 (0.14–0.24)	0.15 (0.09–0.19)	0.05 (0.03–0.07)	0.06 (0.05–0.12)	0.001 *

Method: median (IQR) per group; Kruskal–Wallis across the four groups per vitamin. * omnibus *p* < 0.05. n: CON 7, APAP 5, APAP+MG 9, APAP+LIC 8. Units: mg/kg (ppm). Vitamin A: partial restoration (model lowers A, APAP 6.80 vs. CON 11.79, *p* = 0.018; microgreens raise it, MG 9.54, MG-vs.-APAP trend *p* = 0.083). Vitamin D omnibus not significant (*p* = 0.469). Vitamin C is elevated with injury and B12 lower in treatment arms, non-restorative directions; report panel in full rather than selectively.

**Table 4:** Alpha diversity, Aitchison PERMANOVA, and PERMDISP by group and intestinal site.

Site	Group	Shannon, Median (IQR)	Observed Lineages, Median (IQR)	*n*	KW *p*
Duodenum	Model	1.34 (0.80–2.60)	190 (178–216)	10	0.563
	Microgreens	0.91 (0.87–1.24)	190 (155–198)	9	
	Licorice	0.92 (0.83–2.52)	196 (183–246)	7	
Jejunum	Model	1.26 (0.96–1.36)	198 (181–205)	9	0.090 †
	Microgreens	0.71 (0.40–1.03)	174 (170–192)	10	
	Licorice	0.74 (0.66–1.18)	203 (171–226)	11	
Ileum	Model	0.71 (0.66–1.54)	199 (169–249)	9	0.202
	Microgreens	0.68 (0.39–1.07)	187 (163–199)	8	
	Licorice	1.19 (0.83–2.07)	229 (184–249)	10	
Colon	Model	2.12 (1.78–3.13)	263 (227–279)	9	0.193
	Microgreens	3.68 (3.52–3.71)	282 (272–287)	9	
	Licorice	3.70 (1.38–3.83)	297 (225–307)	8	

Method: median (IQR) of Shannon diversity on the merged genus-level lineage table (655 lineages × 109 mucosal–luminal samples; faeces excluded). Kruskal–Wallis within each site. † 0.05 ≤ *p* < 0.10. Interpretation: no site reached *p* < 0.05; jejunum at trend level. The colonic difference (Model ≈ 2.1 vs. treated arms ≈ 3.7) is accompanied by wide dispersion and is reported as a non-significant trend. Median (IQR) Shannon diversity; Kruskal–Wallis within each site (no site *p* < 0.05). PERMANOVA significant at every site (R^2^ 0.15–0.22, largest in the colon); PERMDISP non-significant in the colon (*p* = 0.641) but significant in the ileum (*p* = 0.049).

## Data Availability

Raw sequencing data from this study have been deposited in the NCBI Sequence Read Archive under accession number PRJNA1498846.

## Supplementary Materials

The following supporting information can be downloaded at: https://www.mdpi.com/article/10.3390/metabo16100698/s1, Figure S1: Alpha diversity (Shannon) by intestinal site; Figure S2: Effect of literature-informed taxonomic filtering on consensus differentially abundant genera; Table S1: Sequencing and quality-control summary; Table S2: Serum metabolite contrasts; Table S3: Contaminant read fraction by group; Table S4: Consensus differentially abundant genera; Table S5: Top microbiome–metabolome integration pairs; Table S6: Lineages removed by the taxonomic filter; Supplementary Methods: ARRIVE 2.0 checklist.
