# Supplementary material for: Colon-Specific Gut Microbiome Remodelling Is Associated with Host Metabolism in Paracetamol-Induced Liver Injury in Rats
Source: Metabolites. 2026 Sep 22;16(10):698. doi: 10.3390/metabo16100698 (PMC13706715; doi:10.3390/metabo16100698)
Supplement: Supplementary file 1 [file metabolites-16-00698-s001.zip › ARRIVE_2.0_checklist.pdf]

ARRIVE 2.0 Reporting Checklist

Manuscript: *"Colon-specific gut microbiome remodelling supports host metabolism in paracetamol-induced liver injury in rats"*

Guideline: Percie du Sert N et al. The ARRIVE guidelines 2.0. Br. J. Pharmacol. 2020. This version is re-synchronised against the current manuscript revision; it corrects earlier entries that contradicted the text (16S analysis, statistical methods) and updates items that are now present (abstract, dose, data availability, declarations).

Status key: Reported = adequately covered. **Partial** = present but incomplete. **Not reported** = missing; add before submission.

Item	ARRIVE 2.0 recommendation	Status	How addressed in this manuscript (location & notes)
**THE ESSENTIAL 10 — the minimum required to assess reliability of the findings**			
**1. Study design**	Groups being compared, including control. Experimental unit (animal, litter, cage).	**Reported**	Four groups compared: CON intact control (n = 7), APAP model (n = 10), APAP+MG (n = 11 → 9), APAP+LIC (n = 10); total N = 38 (§2.2; Fig. 1).<br>Experimental unit = individual animal ("animal as the clustering unit", §2.7). Vehicle control (starch) and mechanistic comparator (licorice) both defined.<br>**Note:** endpoints are staggered (CON/APAP terminated after induction; treatment arms after intervention). Keep this asymmetry explicit so reviewers do not read all four arms as concurrent.
**2. Sample size**	Total number of animals and number per group. How sample size was decided; any a priori power calculation.	**Partial**	Totals and per-group n reported: N = 38 (§2.2; Fig. 1).<br>**GAP:** no a priori power/sample-size calculation or effect-size assumption is stated. Add a justification (feasibility/pilot basis, expected effect size). The 12-animal paired-integration subset (§3.5) is already acknowledged as underpowered — state this alongside.
**3. Inclusion & exclusion criteria**	Criteria for including/excluding animals or data; any exclusions and reasons; whether criteria were pre-established.	**Partial**	Exclusions documented: 2 APAP+MG females died and were excluded (n = 11 → 9; §2.2, Fig. 1). Analysis-level n varies by readout (e.g. vitamins: CON 7, APAP 5, APAP+MG 9, APAP+LIC 8).<br>**GAP:** no pre-specified inclusion/exclusion criteria for animals; not stated whether exclusions were pre-established or post hoc.
**4. Randomisation**	How animals were allocated to groups. Steps to minimise confounders (order of treatment/assessment).	**Not reported**	Allocation to four groups is stated (§2.2) but the randomisation METHOD is not described.<br>**Note:** sequencing run is confounded with group (arms sequenced in separate runs; §2.7) — mitigated by CLR-based modelling but not eliminated.<br>Add the allocation method, or state explicitly that allocation was not randomised and how run-vs-group confounding was handled.
**5. Blinding**	Whether investigators were aware of allocation during allocation, conduct, outcome assessment and analysis.	**Partial**	Liver histology was assessed "blind to group allocation" (§2.4).<br>**GAP:** blinding status for dosing, sample handling, NMR/16S outcome assessment and statistical analysis is not described.
**6. Outcome measures**	Clearly defined outcome measures; for hypothesis-driven studies, the primary outcome.	**Reported**	(a) Composite semi-quantitative liver-injury score (0–15, five domains), §2.4; (b) serum ^{1}H-NMR metabolome (28 metabolites) + vitamins, §2.5; (c) 16S microbiota — alpha/beta diversity and differential abundance, §2.6–2.7; (d) body weight, §2.2.<br>Pre-specified primary hypothesis stated (colon-selective remodelling coupled to a serum-metabolome shift; Introduction).<br>**Note:** serum ALT/AST/bilirubin were NOT measured; biochemical magnitude is inferred from prior reports — disclosed transparently in Results/Discussion.
**7. Statistical methods**	Statistical methods for each analysis; experimental unit; assumption checks.	**Reported**	§2.7: Python 3.12 / R 4.3. Kruskal–Wallis + Benjamini–Hochberg (BH); Mann–Whitney U; PERMANOVA (adonis2, 999 permutations, stratified by animal); PERMDISP; PCA/OPLS-DA (ropls, 7-fold CV, 999 permutations);

Item	ARRIVE 2.0 recommendation	Status	How addressed in this manuscript (location & notes)
			Spearman (BH-FDR) with a permutation-tested PLS axis for integration.<br>Consensus differential abundance = Mann–Whitney U on log-relative abundances AND Welch t-test on CLR-transformed abundances, each BH-corrected and intersected at $q < 0.10$.<br>Sex and intestinal site as covariates; animal = experimental/clustering unit. CLR transformation; raw-vs-filtered sensitivity analyses. Significance $q < 0.05$. NMR: 500 MHz, TSP internal standard, water suppression, 128 scans (§2.1/§2.5).<br>**Corrected:** the previous checklist listed "MaAsLin3 / ANCOM-BC2" and "95% bootstrap CIs", which are NOT used in the manuscript; the actual method is the Mann–Whitney + Welch consensus above.
**8. Experimental animals**	Species, strain, sex, developmental stage, weight; source, health status, genetic status, prior procedures.	**Partial**	38 outbred albino Wistar rats, both sexes, ≈ 3 months (≈ 12 weeks), initial body weight 180–360 g (§2.2).<br>**GAP:** animal supplier/source colony, microbiological/health status and any prior procedures are not stated — the source colony is particularly important for a microbiome study.<br>**Note:** "supplied by Astana IT University" in §2.1 refers to the PLANT (microgreen) material, not the animals — do not conflate.
**9. Experimental procedures**	For each procedure: what, how, when, where; enough detail to allow replication.	**Reported**	Model: paracetamol 1500 mg/kg intragastric in starch, five doses on alternate days over 10 days (§2.2). Interventions: 1 mL/day gavage for 14 days (§2.2).<br>**Corrected:** microgreen dose is now specified — ≈ 8.9 mg microgreen solids per mL, ≈ 35 mg solids/kg/day, with co-delivered fructose/citric/ascorbic acid quantified (§2.1). (The earlier "[Z mg]/[Y mL]" placeholder is resolved.)<br>Preparation of test substance and licorice decoction §2.1; sample collection §2.3; histology (formalin fixation, paraffin embedding, H&E) §2.4; 16S (BGI, V3–V4, LotuS2 v2.23 → DADA2 → ASVs, SILVA v138.1, FastTree2) §2.6.<br>**Corrected:** 16S resolves ASVs — no 97% OTU clustering (the previous checklist stated "97% identity OTU clustering", which contradicts §2.6).<br>**Outstanding:** method of euthanasia not specified (§2.3 states only "animals were euthanised"). Minor: verify the ≈ 8.9 mg total vs the itemised 0.61 + 0.28 mg in §2.1 (arithmetic check).
**10. Results**	Per experiment: summary/descriptive statistics per group; effect size and a confidence interval.	**Reported**	§3.1–3.5; Tables 1–4, S1–S6. Descriptive statistics as median (IQR); p and BH-adjusted q values.<br>Effect sizes: differential abundance as $\log_2$ fold-change (§3.4); PERMANOVA R^2 per site; Euclidean distances to the intact centroid.<br>**Note:** $\log_2$ fold-changes are reported, but formal 95% confidence intervals on effect sizes are not given — consider adding CIs, or state their absence.
**THE RECOMMENDED SET — further context that strengthens the report**			
**11. Abstract**	Accurate summary of objectives, methods, results and conclusions.	**Reported**	**Corrected:** a structured abstract is present (Background/Objectives, Methods, Results, Conclusions) with Keywords. (Previously marked absent — the checklist had been built against a methods-only draft.)
**12. Background**	Scientific background and relevance; what is known; the knowledge gap.	**Reported**	Introduction develops the gut–liver axis, prior microbiota-targeted interventions, and the segment-resolution / dispersion gap this study addresses.
**13. Objectives**	Clear objectives or hypotheses being tested.	**Reported**	A single, pre-specified hypothesis is stated (segment-selective colonic remodelling coupled to a serum-metabolome shift; Introduction).

Item	ARRIVE 2.0 recommendation	Status	How addressed in this manuscript (location & notes)
**14. Ethical statement**	Approving ethics committee, approval reference, and relevant legislation/guidelines.	**Reported**	Innovation Center ArtScience Ethics Committee, protocol No. 6-2023, approved 30 May 2024; Directive 2010/63/EU; ARRIVE 2.0 (§2.2).<br>**Minor:** verify internal consistency of protocol number (2023) vs approval date (30 May 2024).
**15. Housing & husbandry**	Housing (cage/type), husbandry conditions, welfare-related modifications.	**Partial**	"Standard conditions: 20 °C, 12 h light/12 h dark, food and water ad libitum" (§2.2).<br>**GAP:** cage type, animals per cage, bedding, environmental enrichment, diet details and acclimatisation period not stated — expand.
**16. Animal care & monitoring**	Welfare assessment, humane endpoints, actions to reduce suffering; method of killing.	**Partial**	Body weight recorded at baseline and endpoint (§2.2); two deaths in APAP+MG noted.<br>**GAP:** welfare-monitoring schedule, clinical/humane endpoints, analgesia and method of euthanasia (§2.3) not described.
**17. Interpretation / scientific implications**	Interpretation consistent with objectives; limitations; implications for 3Rs.	**Reported**	Discussion presents transferable principles (spatial localisation; PERMANOVA + PERMDISP to separate a genuine centroid shift from a dispersion artefact) and a Limitations section (pilot framing, underpowered integration, co-delivered constituents, plant-matrix confound).
**18. Generalisability / translation**	How findings translate to other species/systems; relevance to humans.	**Partial**	Clinical relevance of paracetamol hepatotoxicity is established; Discussion frames the methodology as transferable.<br>**GAP:** rat → human translation caveats and the single-model limitation could be made more explicit.
**19. Protocol registration**	Whether a protocol was prepared/registered in advance and where; or state none exists.	**Not reported**	No pre-registration or protocol DOI is given. If none exists, add an explicit "not registered" statement.
**20. Data access**	Where study data and materials can be accessed.	**Reported**	**Corrected:** a Data Availability Statement is present; raw 16S data deposited in the NCBI Sequence Read Archive, accession PRJNA1498846. (Previously marked absent.)<br>**Note:** consider also stating access to the serum ^{1}H-NMR/metabolome data.
**21. Declaration of interests**	Conflicts of interest; sources of funding and the funder's role.	**Reported**	**Corrected:** Conflicts of Interest, Funding (Committee of Science, MSHE RK; Grant No. BR24992852) and Author Contributions are all present. (Previously marked absent.)<br>**Minor:** confirm that the cited grant (title referencing a "Smart City Digital Ecosystem") is the correct funding source for this hepatology/microbiome study, or add a line clarifying the link.

Priority gaps to resolve before submission

1. Randomisation & confounding (Item 4): describe the allocation method, or state non-random allocation and how run-vs-group confounding was handled.

2. Sample size (Item 2) and inclusion/exclusion (Item 3): add a pilot/feasibility justification and state whether criteria were pre-established.

3. Blinding beyond histology (Item 5): state blinding (or its absence) for dosing, NMR/16S assessment and analysis.

4. Animal reporting (Item 8) and welfare (Items 15–16): add supplier/source colony and health status; expand housing/husbandry; specify humane endpoints, analgesia and the method of euthanasia (§2.3).

5. Protocol registration (Item 19): add a "not registered" statement if applicable.

6. Consistency checks: euthanasia method (§2.3); the ≈ 8.9 mg vs 0.61 + 0.28 mg arithmetic (§2.1); protocol No. 6-2023 vs approval date 2024; grant relevance (Item 21).

Note on corrections vs the previous checklist: the earlier version described 97% OTU clustering and MaAsLin3/ANCOM-BC2 with bootstrap CIs, and marked the abstract, dose, data-availability and declarations as missing. Those entries did not match the current manuscript and have been corrected here so the checklist can be submitted without contradicting the paper.
